# Different neural activations for an approaching friend versus stranger: Linking personal space to numerical cognition

**DOI:** 10.1002/brb3.1613

**Published:** 2020-04-27

**Authors:** Orly Rubinsten, Nachshon Korem, Anat Perry, Miri Goldberg, Simone Shamay‐Tsoory

**Affiliations:** ^1^ Edmond J. Safra Brain Research Center for the Study of Learning Disabilities The University of Haifa Haifa Israel; ^2^ Department of Learning Disabilities The University of Haifa Haifa Israel; ^3^ Department of Psychiatry Yale University School of Medicine New Haven CT USA; ^4^ Department of Psychology The Hebrew University of Jerusalem Jerusalem Israel; ^5^ Department of Psychology The University of Haifa Haifa Israel

**Keywords:** dyscalculia, event‐related potential, social space, spatial processing

## Abstract

**Introduction:**

Typically, humans place themselves at a preferred distance from others. This distance is known to characterize human spatial behavior. Here, we focused on neurocognitive conditions that may affect interpersonal distances. The current study investigated whether neurocognitive deficiencies in numerical and spatial knowledge may affect social perception and modulate personal space.

**Method:**

In an event‐related potential (ERP) study, university students with developmental dyscalculia (DD) and typically developing control participants were given a computerized version of the comfortable interpersonal distance task, in which participants were instructed to press the spacebar when they began to feel uncomfortable by the approach of a virtual protagonist.

**Results:**

Results showed that students with deficiencies in numerical and spatial skills (i.e., DD) demonstrated reduced variability in their preferred distance from an approaching friend. Importantly, DD showed decreased amplitude of the N1 wave in the friend condition.

**Conclusion:**

These results suggest that people coping with deficiencies in spatial cognition have a less efficient allocation of spatial attention in the service of processing personal distances. Accordingly, the study highlights the fundamental role of spatial neurocognition in organizing social space.

## Graphical abstract

We found that even this seemingly resilient group of young adults with dyscalculia who are enrolled in university display abnormal neuro‐physiological functioning suggestive of a less efficient allocation of spatial attention in the service of processing distances in everyday activities. This deficient attention mechanism leads to abnormal choices of preferred distance from others, mainly from friends, which may have an effect on reciprocal social behavior.

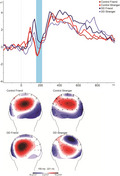

## 1 INTRODUCTION

Personal space is defined as the area that individuals maintain around themselves, into which others cannot intrude without arousing discomfort (Hayduk, 1978). It is significant not only for social relationships, but also as a mechanism that defends the body surface (Graziano & Cooke, 2006). Recent claims in modern science stress interactions between knowledge, perception, action, body, and the environment (Barsalou, 2008; Fischer, 2012). Here, we ask whether deficiencies in knowledge, specifically in numerical and spatial knowledge, may affect how humans act in everyday events in which they must reach a decision as to their preferred personal space. In the numerical cognition field, the metaphor frequently used to describe spatial representation of numbers is the mental number line (MNL), on which numbers are represented in an analogical format, allowing for a proficient processing of numerical quantities (Newcombe, 2002). This assumes that numbers, as symbolic representations of magnitudes and distances, are mapped on a line and are neutrally involved with activation of the intraparietal sulcus (IPS) (for review, see Kaufmann, Wood, Rubinsten, & Henik, 2011). This metaphor is consistent with the distance effect, first reported by Moyer and Landauer (1967), which refers to the negative correlation between the response time (RT) involved in the comparison of two numbers and the numerical distance between them. The distance effect is a behavioral output of a cognitive function, whereby it is harder to discriminate between two numbers that are numerically close than between numbers that are numerically distant. The MNL is typically oriented in space from the left (low numbers) to the right (high numbers) (Dehaene, Bossini, & Giraux, 1993).

During development, people acquire extensive knowledge about numbers (e.g., Landerl, 2013), number sense (Halberda, Ly, Wilmer, Naiman, & Germine, 2012), cardinality and ordinality (e.g., Lyons, Price, Vaessen, Blomert, & Ansari, 2014), and the mental number line (Cicchini, Anobile, & Burr, 2014; e.g., Link, Nuerk, & Moeller, 2014; Rouder & Geary, 2014), all of which are tightly linked to information about distances and space (e.g., Goldfarb, Henik, Rubinsten, Bloch‐David, & Gertner, 2011). For example, Bugden and Ansari (2015) investigated the links between visuospatial working memory and the mental number line by evaluating the approximate number system in 30 children, 15 of them diagnosed with a persistent mathematical disability termed Developmental Dyscalculia (DD; mean age 12). Specifically, they used a nonsymbolic discrimination task (based on Halberda, Mazzocco, & Feigenson, 2008, in which participants had to estimate if there are more blue or yellow dots on the screen) and showed that in the DD group (*r* = −.57), visuospatial working memory skills predict individual differences in the acuity of the mental number line (2015). Another example of a link between the visuospatial working memory and numerical knowledge was demonstrated by Träff, Olsson, Östergren, and Skagerlund (2016), who have found a DD deficit profile that entails both impaired nonsymbolic number processing and impaired visuospatial working memory capacity Hence, here, we wish to investigate whether other non‐numerical experiences that are closely related to the perception of space (e.g., personal distances) may be affected by the intact or deficient development of numerical knowledge, as in cases of developmental dyscalculia (DD).

### 1.1 Personal space

Personal space is thought to be important for the regulation of an individual's interactions with others in interpersonal, as well as in cultural, contexts (e.g., Aiello, Nicosia, & Thompson, 1979; Albert & Dabbs, 1970; Feeney, 1999; Kaitz, Bar‐Haim, Lehrer, & Grossman, 2004; Lloyd, 2009; Sinha & Mukherjee, 1996). For example, Perry and colleagues (Perry, Rubinsten, Peled, & Shamay‐Tsoory, 2013) found that adults coping with social anxiety report feeling discomfort earlier than others in social engagements, and hence, choose to remain further away from other people. In addition, socially anxious individuals showed attenuated P1‐N1 complex. Thus, they had different brain activation in response to figures approaching them.

We must note that there is currently a scientific debate on whether peripersonal action‐space and interpersonal social space refer to similar or different physical distances (Iachini et al., 2016). Peripersonal space is defined as a multisensory visuomotor area, within which one can reach objects in the here and now (Holmes & Spence, 2004). However, recent findings suggest that interpersonal and peripersonal space share a common motor nature, and are similarly sensitive to social aspects (Iachini, Coello, Frassinetti, & Ruggiero, 2014; Iachini et al., 2016). Accordingly, in the current paper, we use the term “personal space” and define it as the distance that people maintain between themselves and others.

Importantly, the type of people (e.g., friend vs. stranger) in one's personal space has a significant effect, not only on one's social relationships but also on the development of efficient protective mechanisms (Graziano & Cooke, 2006). For example, the degree of cognitive closeness (e.g., friend vs. stranger) was found to affect the level of empathy in social situations: The cognitively closer the protagonist (e.g., a friend), the higher the level of empathy (Aron, Aron, Tudor, & Nelson, 1991; Meyer et al., 2012). Empathy is not only essential for adaptive social and moral development (Jolliffe & Farrington, 2004), but also in helping people avoid engaging in potentially harmful behavior, and in alleviating the suffering of others (Eisenberg & Miller, 1987). Thus, the ability to regulate personal space, and to differentially regulate the space around a friend versus a stranger, is of great importance. A deficiency in this ability might be critically detrimental to human development and survival.

Typically, in cases of intact development, one's personal distance from a variety of friends should be highly diverse (compared to one's distance from a stranger). Hall (1966), for example, distinguishes between 3 different personal distances from friends: intimate space, in which the individual can feel the warmth of another person's body (up to 45 cm); personal space, in which the individual can directly interact with the other (up to 1.2 m); and social space, in which the individual can work or meet together (up to 3.6 m). In contrast, strangers typically remain only in the public space (1.5 m or more), where the individual has no physical engagement with other people.

In addition, Perry et al. (2013) showed that typically developing adults allow friends to get closer to them, as compared to strangers. In another study, Perry and colleagues (Perry, Levy‐Gigi, Richter‐Levin, & Shamay‐Tsoory, 2015) found that the higher the social anxiety is, the greater the variance in interpersonal distance preferences in individuals with autistic spectrum disorder. Among participants with nonclinical social anxiety, there was a distance estimation bias, so that participants estimated the interpersonal distance from strangers as shorter than it really was. Moreover, a recent study found an association between the distance estimation bias and the preferred distance from a stranger, so that participants with preference for a greater distance also estimated this distance as shorter (Givon‐Benjio & Okon‐Singer, 2020).An important neglected factor that may contribute to a distance estimation bias or a variance in interpersonal distance preferences is one's level of spatial skills. The idea behind this current hypothesis is that if one is deficient in processing spatial information, s/he would prefer a specific distances from others, mainly from friends who are typically allowed to approach closer (Perry et al., 2013), and this is, we assume, to avoid being over stimulated and uncomfortable. Hence, in the case of approaching friends, people with deficiencies in spatial processing, such as those with DD, may show a reduced variability in their preferred distance from friends. Hence, the current study aims to investigate the variance in different estimations of the personal space of friends versus strangers, when spatial and numerical processing is deficient (i.e., among DD).

### 1.2 Developmental dyscalculia and numerical/spatial skills

Developmental dyscalculia is reflected in several different numerical dysfunctions, among which is the ability to estimate and compare nonsymbolic numerical quantities (e.g., dot arrays comparison: Piazza et al., 2010; Bulthé et al., 2019; dot arrays enumaration: Estévez‐Pérez, Castro‐Cañizares, Martínez‐Montes, & Reigosa‐Crespo, 2019; ordinality: Rubinsten & Sury, 2011) and to process numbers symbolically (e.g., De Smedt & Gilmore, 2011; Geary, Hamson, & Hoard, 2000; in Arabic notation—Furman & Rubinsten, 2012; Rousselle & Noël, 2007; for review see Kaufmann et al., 2013; Stock, Desoete, & Roeyers, 2010), resulting in a deficient distance effect. For example, Price and colleagues (Price, Holloway, Vesterinen, Rasanen, & Ansari, 2007) found differences between DD and controls in the distance effect during symbolic comparisons in an fMRI study. They found weak IPS activation in DD children compared to controls when comparing nonsymbolic numerical stimuli. Moreover, Landerl and Kölle (2009) found that 8‐ to 10‐year‐old DD children were slower than controls in judging the numerical distance of two digits (i.e., 3–5) with medium effect size (*η* ^2^ = 0.04), but not the physical size (i.e., 3–3). Mussolin, Mejias, and Noël (2010) found that 10‐ and 11‐year‐old children with DD showed a larger distance effect in both symbolic and nonsymbolic numerical comparisons. Also, using the event‐related potentials (ERPs) methodology, Soltesz and colleagues (Soltesz, Szucs, Dekany, Markus, & Csepe, 2007) found that, compared to controls, adolescents with DD show no late event‐related brain potentials (ERPs) distance effect between 400 and 440 ms on right parietal electrodes when comparing Arabic numerals. Such findings suggest that the processing of numerical information, and of representations of the distances between them, may be abnormal in DD.

Collectively, the behavioral and electrophysiological evidence points to a deficient representation of the mental number line, in which quantities are spatially mapped based on distances, as one of the possible outcomes or sources of DD. Recent findings suggest that such a deficient mental number line is strongly linked to visuospatial working memory skills (e.g., Bugden & Ansari, 2015; Rubinsten & Henik, 2009; Szucs, Devine, Soltesz, Nobes, & Gabriel, 2013), and hence, DD may indicate deficient spatial skills.

Although characterizing and remediating DD (Kaufmann et al., 2013; Szűcs & Goswami, 2013) are clearly critical, the primary interest for cognitive neuroscience is to learn how conditions such as DD can inform existing cognitive theory. The current study specifically aims to investigate whether other non‐numerical spatial experiences may be affected by the deficiency in spatial representations of numerical information. Indeed, it has been shown that the process of extracting numerical information is tightly linked to different types of non‐numerical information processing that are influenced by environmental and spatial experiences. For example, evidence shows that the mental number line proceeds from left to right. However, Holmes and Lourenco (2012) presented numerical tasks which were primed by a story that allowed participants to think about numbers in the context of referring to floors in a multi‐story building. Results showed a vertical mental number line (instead of left to right), oriented from bottom (i.e., low numbers) to top (i.e., high numbers). These results provide strong evidence for an association between spatial representations of numbers, personal experiences, and non‐numerical spatial contexts (see also Fischer, 2008) (Hartmann, Gashaj, Stahnke, & Mast, 2014).

Here, we specifically wish to investigate whether numerical and spatial deficiencies in the case of DD also affect non‐numerical spatial representations, such as those that require the processing of personal distances and space in everyday situations. Indeed, spatial cognition (i.e., deficient in DD; e.g., Bugden & Ansari, 2015; Rubinsten & Henik, 2009; Szucs et al., 2013) has been strongly linked to social cognition (Schubert & Maass, 2011), as indicated by personal space. In a summary paper, Schubert emphasized that spatial representations are the “…medium of social interactions—the stage of our social life” (Schubert & Maass, 2011 page 1) and argued that social relations take place, among other things, in horizontal distance, and in its change in approach or avoidance (i.e., personal space). Similarly, in reviewing a wealth of scientific work, Tversky (2011) argued that spatial cognition serves as the basis for social thoughts. For example, in two studies, Schnall and colleagues (Schnall, Harber, Stefanucci, & Proffitt, 2008) showed that adult participants accompanied by a friend estimated a hill to be less steep, when compared to participants who were alone both in a real situation (Study 1; *N* = 34 participants) and also during an imagery task (Study 2; *N* = 36 participants).

Yet, to the best of our knowledge, contemporary scientific investigations have not dealt with the question of whether the regulation of personal space requires intact spatial cognitive resources.

### 1.3 The current study and methodological issues in investigations of personal space

Personal space has been studied in different contexts, such as brain networks (Holt et al., 2014) and hormones (Perry, Mankuta, & Shamay‐Tsoory, 2014), that are involved with regulation of personal space, and in the context of individual differences such as attachment style in infancy (Bar‐Haim, Aviezer, Berson, & Sagi, 2002). Also, and with high relevance to the current work, researchers have recently suggested that spatial cognition informs social cognition (Schubert & Maass, 2011). However, to date and to the best of our knowledge, no study has investigated the interaction between existing cognitive representations, such as numerical representations that are based on distances and space, and regulation of personal space.

Both numerical processing (e.g., Soltesz et al., 2007) and spatial attention (e.g., Hillyard, Vogel, & Luck, 1998) have been shown to occur from very early stages (about 100–150 ms poststimulus presentation) of processing in the brain. Hence, measuring event‐related potentials (ERPs) in an interpersonal distance task enables us to better understand the effects of DD on these early stages. The major cognitive component of interest is the ability to be spatially attentive to changes in personal distances. Studies on the topic of attention, and more specifically of spatial attention, have shown that ERP components (P1‐N1) reliably reflect differences in the processing of attended and unattended (e.g., Hillyard et al., 1998), or less attended information (Vogel & Luck, 2000). By recording ERPs to stimuli with changing personal distances, direct evidence can be obtained on the level of processing achieved by these stimuli. The most consistent finding for an attentional effect is the N1 wave (a negative wave peaking about 150 ms after stimulus presentation) (e.g., Haider, Spong, & Lindsley, 1964; Martínez et al., 2006). When a particular location in the visual scene is attended, N1 waves elicited by stimuli at that location are enlarged (e.g., Mangun & Hillyard, 1988), an effect that has been suggested to be a sign of attentional modulation of spatial processing. Accordingly, we hypothesize that participants with a deficient ability to estimate numerical distances (i.e., DD) will also show deficiencies in their ability to regulate their personal space (resulting, e.g., in decreased variability of personal distances from friends), and that this type of deficit in spatial processing will show a biomarker in the form of modulated posterior N1 wave.

In conclusion, we were interested in investigating whether the known deficit of people coping with DD to process numerical distances also appears as a deficit in their ability to regulate their preferred interpersonal distance. Specifically, we aimed to discover whether, due to their numerical deficiencies, young high functioning university students diagnosed with DD are deficient in their ability to spatially attend to their personal space and, hence, struggle to regulate their preferred interpersonal distance from friends or strangers. A deficit in the ability to regulate distances, specifically personal distances, is assumed to result in a modulated posterior N1 wave. Such altered nerurophysiological findings may indicate significant associations between social cognition and spatial perception.

## 2 MATERIALS AND METHOD

### 2.1 Participants

Twenty‐eight young adults participated in the study; 14 of them, previously diagnosed with dyscalculia, comprised the clinical sample, while the remaining 14 comprised the control sample.

Due to excessive noise in EEG raw data (see analysis below, for description of EEG noise), 3 female DD and 2 female control participants were excluded from the data analysis. Hence, the reported results are based on 23 participants (11 DD and 12 controls). Table 1 presents the characteristics of the clinical and control samples.

**Table 1:** Descriptive information and percentile range scores in the selection tasks for the DD and control groups

	Control group	DD group	
Descriptive information			
*N*	12	11	
Gender (M/F)	5/7	0/11	
Age	29 y, 6 m (*SD* = 2 y, 6 m)	30 y, 5 m (*SD* = 4 y, 9 m)	
Mathematics			
Simple calculation (calculation automaticity)‐ACC	31–50	6–10	2.234*
Simple calculation (calculation automaticity)‐RT	50–66	3–5	3.059**
Procedural knowledge‐ACC	76–89	11–12	3.283**
Procedural knowledge‐RT	55–64	6–10	2.892**
Number line positioning‐ACC	43–39	16–18	2.022*
Number line positioning‐RT	66–88	16–21	0.583,NS
Reading			
Text reading‐ACC	58–78	57–76	0.615,NS
Rapid naming‐letters	83–84	78–83	1.624,NS
Rapid naming‐numbers	73–77	44–56	2.017*
Attention (Questionnaire)			
Attention difficulties‐general	38–41	35–41	0.899,NS
Implosive and hyperactive reports	42–62	62–67	0.668,NS
Childhood attention symptoms	45–55	51–60	0.046,NS
Adolescent attention symptoms	62–67	60–65	0.11,NS

Note

Standard deviations are shown in parentheses.

Abbreviations: ACC, accuracy; m, months; RT, reaction time;y, years.

Significance independent sample *t* test (one tails) *T *(21) = *p* < .05, **p < .01.

All participants reported normal or corrected‐to‐normal visual acuity, and no history of psychiatric or neurological disorders, as confirmed by a screening interview.

Fourteen developing adults (see Table 1) were recruited through advertisements distributed on the university campus. Additionally, 14 adults who had been diagnosed with DD (see Table 1) were recruited through a search in the diagnosis database of the Haifa University clinic for learning disabilities (students diagnosed at the clinic are typically asked to sign a waiver that allows their test scores to be used for research purposes). In addition, since the database did not produce a sufficient number of participants, advertisements were posted on the university campus as well as at nearby colleges. To confirm the diagnosis, we used a standardized computerized assessment tool: the “Israeli Learning Function Diagnosis System” (entitled “MATAL” in Hebrew) for high school and higher education students (National Institute for Testing & Evaluation—NITE. For more details, see e.g., Kennet‐Cohen, Bronner, Ben‐Simon, & Intrator, 2008 as well description of the MATAL below).

Participants gave written consent to participate in the experiment and were paid about USD 15 for their participation.

### 2.2 Classification and assessment criteria

All participants were classified as controls or DDs using the “Israeli learning function diagnosis system” (also entitled “MATAL” in Hebrew) for high school and higher education students (National Institute for Testing & Evaluation—NITE. For more details, see e.g., Kennet‐Cohen et al., 2008). MATAL was developed by NITE in cooperation with the Israeli Council for Higher Education (CHE), as part of an endeavor to develop policies and procedures for standardizing and regulating the diagnosis of learning disabilities in higher education, thus facilitating the provision of testing accommodations. The MATAL was validated and normed on adults (ages 16–30, with different levels of education) results. Based on the results of the validation study, a prediction model was developed by NITE for the diagnosis of four disabilities. MATAL assessment tools include 20 tests that assess the following skills: reading, writing, numeracy, attention, memory, and visual perception. Of the 20 tests, three (7 performance measures) are used to diagnose numeracy functions: Computational Automaticity (retrieval of simple arithmetic facts), Procedural Knowledge (mastery of basic arithmetic procedures), and Number Sense (number‐line representation). All MATAL tests have been validated and normed (Kennet‐Cohen et al., 2008).

A further selection of potential participants was carried out on the basis of their performance on MATAL reading and attention tests. The attention tests include a MATAL questionnaire based on the DSM IV and the Connors questionnaire (Conners, Erhardt, & Sparrow, 1999), which includes the ability to diagnose past (i.e., childhood and adolescent) symptoms of inattention.

The cutoff inclusion threshold was a score below (for the DD group) or above (for the control group) the 20th percentile in either RT or accuracy (ACC) on the three arithmetic tests. In addition, only participants with no reading or attention deficiencies were included in the study. That is, only those participants who were above the 50th percentile (for both DD and control groups) in the reading tests and above the 40th percentile in the attention tests could participate in the study (see Table 1).

Importantly, a Mann–Whitney *U* test revealed no significant effects for gender (i.e., no differences between males and females) within the control group in any of the tests.

After the EEG experiment, participants completed the Liebowitz Social Anxiety Scale (LSAS) to ensure that they had no social anxiety (and that social anxiety did not act as an interfering variable), since social anxiety was previously shown to be correlated with interpersonal distance preferences (Perry et al., 2013). Specifically, participants completed a computerized version of the LSAS (Liebowitz, 1987), one of the most commonly used and validated clinician‐administered scales for the assessment of social anxiety (Fresco et al., 2001; Heimberg et al., 1999; Mennin et al., 2002). Participants were asked to rate their levels of fear and avoidance of 24 situations on a scale of 0–3. The 24 items were divided into two subscales that address social interaction (11 items) and performance (13 items). No statistical differences were found between the groups in terms of LSAS (see Table 2).

**Table 2:** LSAS scores of the clinical and control samples

	Social interaction (Avoidance)		Performance (Anxiety)		Total Score (LSAS)	
	Mean	*SD*	Mean	*SD*	Mean	*SD*
DD group	17	3.02	18	3.34	36	5.86
Control group	18	3.41	20	3.81	38	7.06
	*T* (21) = 0.287,NS		*T* (21) = 0.173,NS		*T* (21) = 0.244,NS	

### 2.3 Stimuli task and design

**Figure 1:**
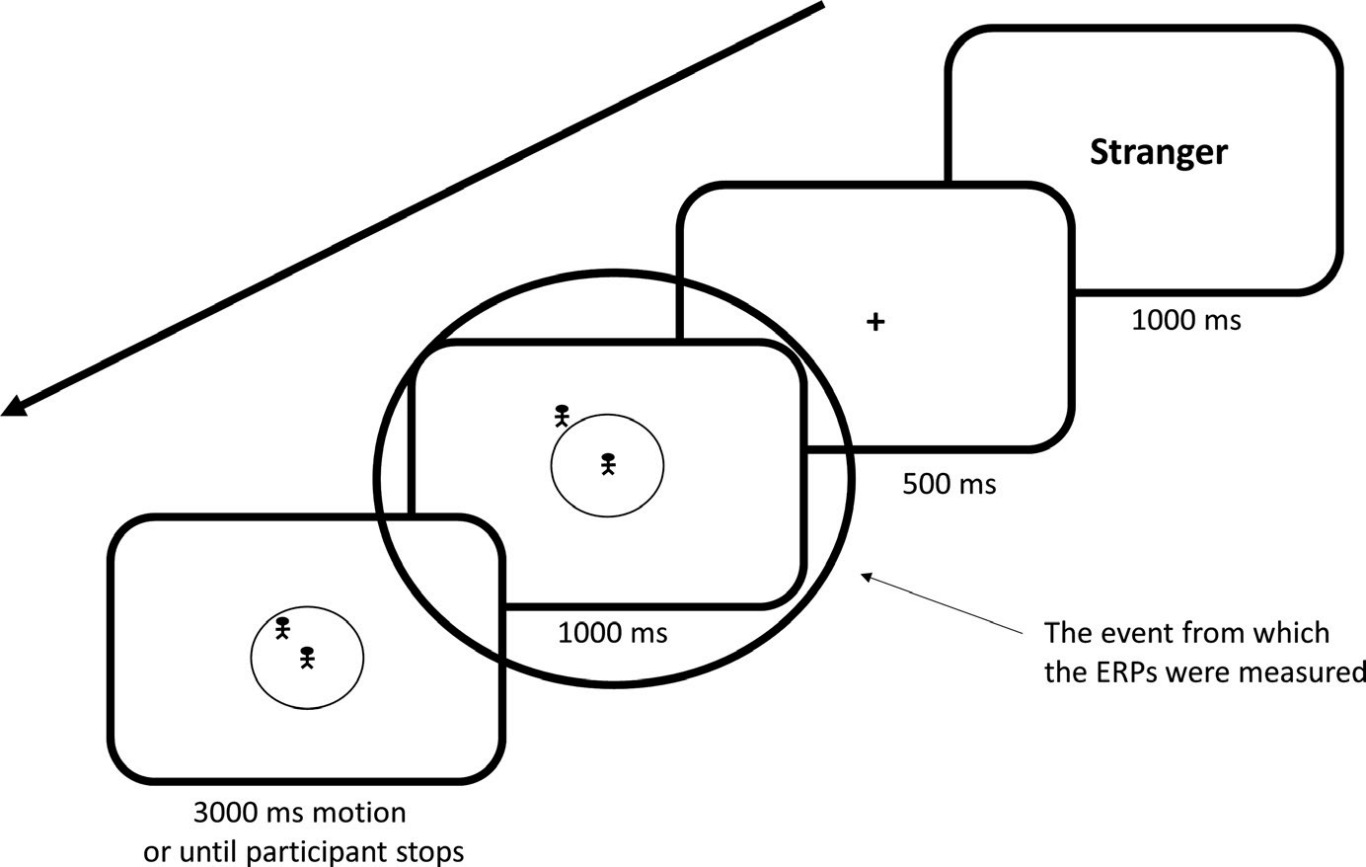
Experimental design

The stimuli used were a modified version of a paper‐and‐pencil validated measure of comfortable interpersonal distance (CID—Duke & Kiebach, 1974; Duke & Nowicki, 1972), in which a circular room is shown, with line figures depicting one's self in the center of the room and a protagonist outside the room. In this modified computerized version of the CID, we further defined the protagonist entering the room as either a close friend, a stranger, a ball, or an artificial figure presented as a “griple” and composed of mixed parts of the human line figure. Based on previous studies (Perry et al., 2013) and following the current research questions, only the friend and stranger protagonists were retained for analysis. The participant saw a word depicting the type of the protagonist who would enter the room for 1,000 ms, a fixation point for 500 ms, and then a still picture (1,000 ms) of the circular room with a character in the center and the approaching protagonist in one of the eight entrances. This was followed by a 3,000 ms animation in which each different protagonist approached the center of the circle. Since the image is two dimensional, the approaching protagonist was always in front of the person (i.e., coming from any of the 8 entrances) located in the center of the circular room. As in the original version, the participants were instructed to imagine themselves in the center of the room, and to respond to the virtual protagonist approaching them along a particular radius, this time by pressing the spacebar when they started to feel uncomfortable. The animation stopped after three seconds, when the character and the protagonist collided, or beforehand, at a press of the spacebar (Figure 1). In order to measure ERPs, the 1,000 ms still picture depicting the room with the protagonist ready to approach was the crucial “event” for ERP analysis. Responses were computed as the percentage of the remaining distance from the total distance. In contrast to the original paper and pencil version where the responses were spontaneous and fast, the responses in this experiment were less spontaneous, as they were elicited a few seconds after the name of the figure appeared, in order to measure the ERPs without motor interference. In order to enable enough data for ERP analysis, each of the approaching protagonists (4) appeared 56 times (7 repetitions of the 8 radii, collapsed for analysis), giving a total of 224 trials. To note, as in Perry et al. (2013), since only 2 protagonists were studied, only 112 were analyzed. There were two breaks during the experiment, allowing participants to rest. In order to avoid eye movements, the stimuli size was reduced, such that the radius of the circle was 45 mm and the line length was 6 mm. The experiment was presented on a CRT monitor, 60 cm from the participant's eyes, with the circle's diameter creating a visual angle of 8.58°. E‐Prime 2.0 (Psychological Software Tools) was used for stimulus presentation.

### 2.4 Data acquisition and analysis

#### 2.4.1 EEG recording

The EEG analog signals were recorded continuously (from DC with a low‐pass filter set at 100 Hz) from 64 Ag‐AgCl pin‐type active electrodes mounted on an elastic cap (Biosemi, http://www.biosemi.com/headcap.htm), according to the extended 10–20 system, and from two additional electrodes placed at the right and left mastoids. All electrodes were referenced during recording to a common‐mode signal (CMS) electrode between POz and PO3, and were subsequently re‐referenced digitally (see data processing below). Eye movements, as well as blinks, were monitored using bipolar horizontal and vertical EOG derivations via two pairs of electrodes, with one pair attached to the external canthi and the other to the infraorbital and supraorbital regions of the right eye. Both EEG and EOG were digitally amplified and sampled at 512 Hz, using a Biosemi Active II system (www.biosemi.com).

#### 2.4.2 Data processing

Data were analyzed using Brain Vision Analyzer software (Brain Products). Raw EEG data were initially 0.5 Hz high‐pass and 30 Hz low‐pass filtered (24 dB) and re‐referenced offline to the digital average of the 64 electrodes. EEG deflections resulting from eye movements and blinks were corrected using an ICA procedure (Jung et al., 2000). Remaining artifacts exceeding ± 100 μV in amplitude, a voltage step of over 50 μV, or low activity of under 0.5 μV change over 100 ms were rejected.

### 2.5 Statistical analysis

For statistical analysis, independent sample *t* tests, mixed‐design two‐way ANOVA, bivariate and Mann–Whitney *U* test analysis were used as indicated.

### 2.6 Behavioral and ERP analyses

#### 2.6.1 Behavioral analysis

Similar to behavioral analyses in Perry et al. (2013) and following the current research questions, here too a mixed‐design two‐way repeated‐measure ANOVA (Group × Protagonist) was conducted on the average distance in which each group stopped the friend and stranger protagonists only. The ball and “griple” trials were discarded. In addition, we examined the variability of each individual's responses (i.e., distances) separately for friends and strangers. For this purpose, we conducted a mixed‐design two‐way repeated‐measure ANOVA (Group × Protagonist) on the standard deviations (*SD*) of participants' responses (i.e., distances) to the protagonists. Also, a Pearson correlation was calculated between the CID scores and the total LSAS scores. A total of only 2 trials were omitted from analysis since the participants in these two trials stopped the protagonist at the maximum distance (the trials are from 2 different DD participants—one trial per each DD).

Finally, there were also several significant statistical links between numerical skills and the preferred distance. Specifically, there was a significant correlation between average distance and RTs in procedural knowledge (*r* = −.606, *p* < 005) and number line positioning (*r* = −.609, *p* < .05) but only in the control group (DD: *r* = −.284, NS; *r* = −.398, NS). In the DD group, there was a significant correlation between average friend distance and simple calculation ACC (*r* = −.737, *p* < .01).

### 2.7 ERP analysis

ERPs were analyzed in two separate analyses of a mixed‐design three‐way ANOVA [Group (2) X Protagonist (2) X Electrode (4)] on the average one‐second segment trials of the protagonists. One analysis included Latencies as within subject variables and the other included amplitude. The averaged segments were baseline corrected to 200 ms before stimulus onset. N1 component was calculated under the lateral‐occipital electrodes (O1, O2, PO7, and PO8) (e.g., Jepma, Wagenmakers, Band, & Nieuwenhuis, 2009; Pourtois, Grandjean, Sander, & Vuilleumier, 2004 who all used similar lateral‐posterior bund of electrodes to study P1/N1) (see also Di Russo, Martínez, & Hillyard, 2003; Luck & Yard, 1995).

For each participant, the peak of the N1 was determined as the most negative peak between 150 and 220 ms. Subsequent visual scrutiny ensured that this value represented real peaks rather than end points of the epoch (see also Campanella et al., 2002; Foti, Hajcak, & Dien, 2009).

### 2.8 Ethics statement

The recruitment, payment, tasks, and overall procedure were authorized by the Research Ethics Committee of Haifa University and by the Research Ethics Committee of the Faculty of Education. All methods and experimental protocol were approved by the Research Ethics Committee of Haifa University (#123/09) and by the Research Ethics Committee of the Faculty of Education (#144/14) and were carried out in accordance with the approved guidelines. In addition, informed consent was obtained from all participants.

## 3 RESULTS

### 3.1 Behavioral results

A repeated‐measure mixed‐design ANOVA of the mean preferred distances between the participants and protagonists revealed a significant effect for the protagonist [*F* (1, 21) = 48.775, *p* < .001, *η* ^2^ = 0.699; See Table 3], whereby participants stopped the friend [*M* = 14.304, *SD* = 7.989] much closer to them than they did the stranger [*m* = 46.451, *SD* = 19.55]. Other than that, there were no effects for group [Control: *M* = 33.764, *SD* = 24.934, DD: *M* = 26.81. *SD* = 18.024; *F* (1, 21) = 2.998, NS], nor interaction between group and protagonist [*F* (1, 21) = 1.779, NS].

**Table 3:** Mean stopping distance of protagonist

	Friend		Stranger	
	Mean	*SD*	Mean	*SD*
DD group	13.372	8.913	39.639	15.283
Control group	14.833	7.402	52.696	21.521

A repeated‐measure mixed‐design ANOVA of the *SD* of the preferred distance from the protagonist revealed a marginal significant effect for protagonist [*F* (1, 21) = 3.503, *p* = .075, *η* ^2^ = 0.143], and a significant effect for group [*F* (1, 21) = 6.005, *p* < .05, *η* ^2^ = 0.222], whereby DD participants were more consistent with regard to the point at which they stopped the protagonist [*m* = 7.025 *SD *= 0.779] than were control participants [*m* = 9.187, *SD *= 0.745]. No significant interaction was found [*F* (1, 21) = 0.298, NS].

**Figure 2:**
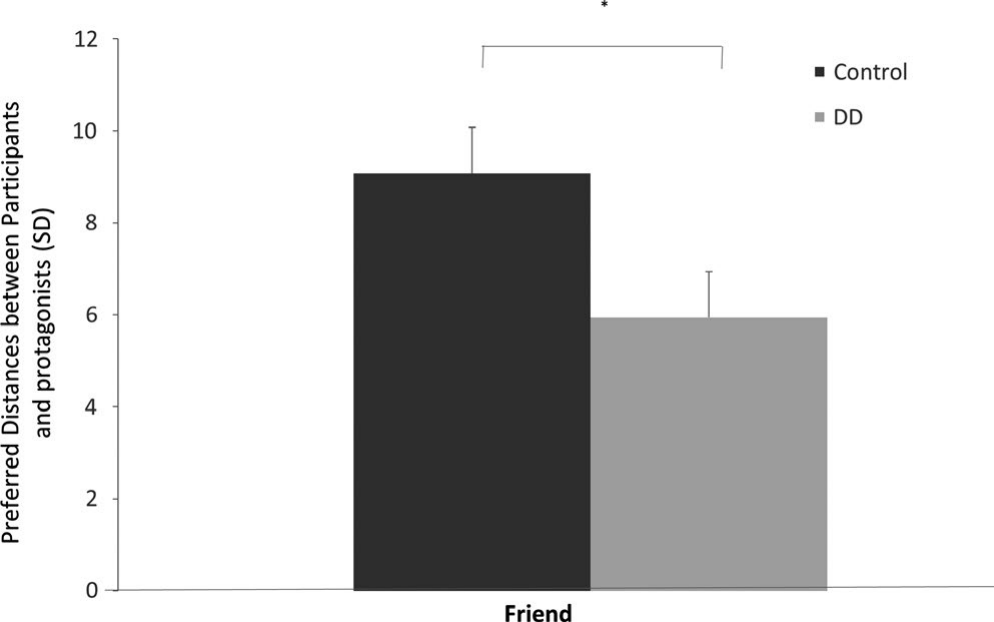
Behavioral findings (standard deviation as a dependent variable): Friend versus stranger in each group separately *p < .05

Despite the insignificance of the interaction, and due to the ERP results (see Electrophysiological results in the next section), we analyzed the two protagonists in each group separately. An independent samples *t* test of the distance *SD* in the friend condition revealed a significant effect (*T* (21) = 2.123, *p* < .05), as DD showed a reduced variability in their preferred distance from an approaching friend [*m* = 5.939 *SD* = 2.576] than did control participants [*m* = 9.071, *SD* = 4.221]. No such effect was present in the stranger condition (*T* (21) = 1.62, *p* = .12; See Figure 2).

No significant correlations were found between the LSAS scores and the average distance at which the participants stopped each of the protagonists.

Note also that a Mann–Whitney U test revealed no significant differences between male and female controls in neither the CID protagonists nor the LSAS score.

### 3.2 Electrophysiological results

**Figure 3:**
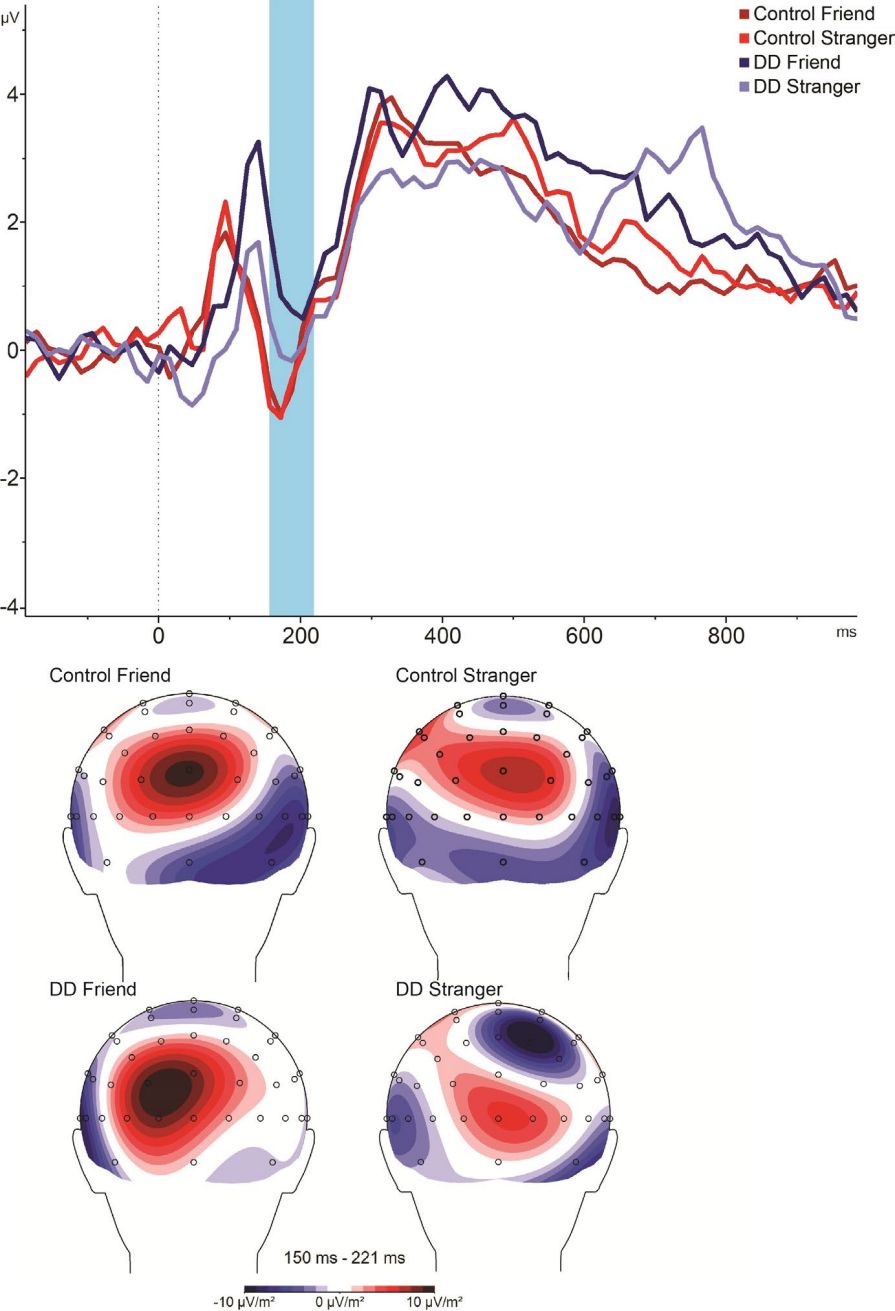
N1 wave (in each group, separately for friend and for stranger)

Repeated‐measures ANOVAs of mean amplitudes and latency of parieto‐occipital electrode groups in the 150–220 ms poststimulus time windows (N1) revealed a significant effect for groups on both amplitude and latency [amplitude: *F* (1, 21) = 6.296, *p* < .05, *η* ^2^ = 0.231; latency: *F* (1, 21) = 4.515, *p* < .05, *η* ^2^ = 0.177], as the DD showed delayed lower (less negative) amplitudes. In addition, a significant effect for protagonist on amplitude [amplitude: *F* (1, 21) = 5.168, *p* < .05, *η* ^2^ = 0.198; latency:* F* (1, 21) = 0.209, NS] was found. See Figure 3.

Most importantly, a significant interaction on amplitude only was found between group and protagonist [amplitude: *F* (1, 21) = 6.411, *p* < .05, *η* ^2^ = 0.234; latency: *F* (1, 21) = 2.167, NS].

Post hoc tests revealed that the source of the interaction was a significant difference in amplitude between the control group and DD group in the friend condition [*F* (1, 21) = 10.32, *p* < .01, *η* ^2^ = 0.33], as the DD group showed a significantly less negative peak (*M* = 0.843, *SD* = 2.216) compared with control (*M* = −2.121, *SD* = 3.031), while such a significant difference was absent in the stranger condition [*F* (1, 21) = 2.344, NS; control: *M* = −2.039, *SD* = 2.837; DD: *M* = −0.657, *SD* = 2.097].

## 4 DISCUSSION

The current study aimed to investigate the neurophysiological correlates of personal distance regulation among university students with DD during a computerized version of the comfortable interpersonal distance task (CID—Duke & Kiebach, 1974; Duke & Nowicki, 1972). As hypothesized, we found that, compared to typically developing controls, university students with DD showed different behavioral patterns: DD demonstrated a reduced variability (i.e., they showed more consistent performance) in their preferred distances from approaching friends. Normally, personal distances between a person and his/her different friends should be highly diverse (compared to a distances from strangers) (e.g., Hall, 1966). Hence, current results suggest that participants with deficiencies in processing spatial information (DD) prefer a specific distances from others, mainly from friends who are typically allowed to approach closer (Perry et al., 2013; as well as current results—see the significant difference between friend vs. stranger beyond Groups), and this is, possibly, to avoid feelings of distress. Thus, in the case of approaching friends, people with DD showed a reduced variability in their preferred distance, which suggests that DD participants are deficient in regulating personal space.

In accordance with behavioral findings, electrophysiological data showed that, compared to controls, DD demonstrated a delayed and reduced (less negative) N1 wave. This reduced N1 amplitude appeared mainly in the friend condition.

This posterior *reduced* early neural component (N1) in the DD group, a wave (N1) that has been typically associated with perception and spatial attention (e.g., Hillyard et al., 1998) in the DD group, suggests that regulation and perception of personal space is strongly linked to basic numerical and spatial neurocognitive functioning. Importantly, it had been shown that, as attention decreases, the amplitude of the N1 decreases, suggesting that the amplitude of the N1 is closely linked to levels of attention (e.g., Haider et al., 1964) (Van Voorhis & Hillyard, 1977). Against this background, the current significantly decreased N1 wave elicited in the DD group by an approaching friend may be related to deficient social‐spatial mechanisms, that is, to not devoting intact spatial resources to the social situation.

Clearly, human ability to relate differently to friends vs. strangers has important personal, as well as social outcomes (Aron et al., 1991; Meyer et al., 2012). The physiological and behavioral findings suggest that DD is deficient in their ability to estimate and to decide upon the most comfortable personal space for them. Hence, DDs' basic cognitive deficiencies in numerical and spatial cognition have an ecological effect on everyday activity that requires regulation of personal space.

Importantly, spatial cognition has previously been strongly linked to social cognition (e.g., Parkinson & Wheatley, 2013; Proulx, Todorov, Taylor Aiken, & de Sousa, 2016). However, contemporary scientific investigations have not dealt with the question of whether the regulation of personal space requires intact spatial cognitive resources. The novel contribution of the current study is that by using conditions such as DD to inform existing neurocognitive investigations of social cognition, we point to a significant link between spatial processing and social space. We show that the regulation of personal space requires intact spatial cognitive resources, and we highlight the fundamental role of spatial cognition in organizing social space.

In the following, we discuss the ERP signature.

### 4.1 Posterior N1

The modulated N1 attentional effect in the DD group may indicate that cognitive processes are engaged to different degrees by the two groups of participants. Accordingly, the posterior N1 wave, which suggests “early selection” by the attention cognitive system, indicates here that sensory processing is affected by deficient attention to personal distance prior to the completion of perceptual analyses.

Perceptual analysis depends mainly on (a) the intensity of sensory stimulation and (b) top‐down attention, which enhances sensory processing (Dehaene, Changeux, Naccache, Sackur, & Sergent, 2006).

Regarding the intensity of the sensory stimuli, it is important to note that N1 is typically elicited by external stimuli that are strongly influenced by stimulus parameters, such as luminance (Johannes, Münte, Heinze, & Mangun, 1995), spatial frequency (e.g., Hansen, Jacques, Johnson, & Ellemberg, 2011), and depth (i.e., 2 vs. 3 dimensional stimulus, e.g., Omoto et al., 2010). However, the current personal space effect has been found to resist manipulation of the nonpersonal spatial parameters of the display (e.g., there are no differences in the physical appearance of friends and strangers), thus evading simple explanations in terms of density or area.

We also suggest here that spatial attention to personal distance is deficient in DD and, hence, limits perception and regulation of personal space. It has been shown that responses to stimuli presented at attended locations are augmented, and further processing of these stimuli will therefore be enhanced (Hillyard et al., 1998). More specifically, the N1 was found to represent the spatial orienting of attention to the relevant stimulus (Luck, Heinze, Mangun, & Hillyard, 1990). Here, the N1 wave was found to be delayed and less negative in people diagnosed as DD, suggesting an inability to properly attend to their personal space and, hence, to perceive it.

As mentioned, the degree of cognitive closeness (e.g., friend vs. stranger) is correlated with the intensity of empathy (i.e., thinking about the mental states and intentions of others) in social situations (Aron et al., 1991). In this context, it is reasonable to argue that processing information (e.g., social space) that is related to a friend requires augmented attentional resources, compared to processing information that involves a stranger. That is, for the typically developing population, an approaching friend requires more attentional resources than an approaching stranger. However, for people with a deficient ability to estimate distances (i.e., DD), social situations that require enhanced spatial attention, such as regulating a friend's social space, result in ineffective allocation of attention for evaluating the friend's social space. Such a deficit has a biomarker in the form of a modulated N1 wave.

Limitations of this study include the modest sample size and exclusive use of 2D animation of an approaching person. Indeed, Latif and colleagues (Latif, Barbosa, Vatiokiotis‐Bateson, Castelhano, & Munhall, 2014) found that observers use coordination to judge affiliation (friend vs. stranger) between conversing pairs, but only when the perceptual stimuli were restricted to head and face regions. Their findings suggest that for typically developing participants, perception of friends should be more diverse when the perceptual stimuli (e.g., the approaching friends, as in the current study) are not restricted to head and face (as in the current study, in which a very simple 2D animation of a whole body is presented). Future studies should compare between 3D presentations of heads/faces only versus whole body movements. Strengths of this study include the focus on a highly pertinent sample of DD, and the robust examination of electrophysiological associations among symptoms of DD and social cognition.

## 5 CONCLUSIONS

We found that young, relatively high functioning university students with DD show different neurophysiological processing patterns during a task that requires them to estimate their preferred personal distances. It seems very reasonable to suggest that in DDs, spatial attention to social distances is poor, due to their deficiencies in estimating numerical distances (e.g., Bugden & Ansari, 2015; Rubinsten & Henik, 2009; Szucs et al., 2013). As a result, protagonists, and specifically friends, are processed differently inside their DD's social space.

We clearly show that deficiencies in a specific neurocognitive domain, in the current study spatial numerical cognition, interact with everyday activities that are not directly related to this numerical domain. The results of this study, therefore, provide evidence that even this seemingly resilient group of young adults with DD who are enrolled in university, display abnormal neurophysiological functioning, suggestive of a less efficient allocation of spatial attention in the service of processing distances in everyday activities. This deficient spatial mechanism leads to abnormal neural activation related to preferred distance from others, mainly from friends, which may have an effect on reciprocal social behavior. Accordingly, the study highlights the fundamental role of spatial neurocognition in organizing social space.

## CONFLICT OF INTEREST

The authors declare no competing financial interests.

## AUTHOR CONTRIBUTIONS

Orly Rubinsten, Nachshon Korem, Anat Perry, Miri Goldberg, and Simone Shamay‐Tsoory contributed to the development of research questions, methods, running the experiment, statistical analysis, and writing up the paper.

## Data Availability

The data that support the findings of this study are available on request from the corresponding author. The data are not publicly available due to privacy or ethical restrictions.
